# Brain Health Scotland (BHS) – a Pioneering Brain Health Service in Aberdeen, Scotland

**DOI:** 10.1192/bjo.2025.10099

**Published:** 2025-06-20

**Authors:** Tom MacEwan, Poppy Betts, Karen Black, Lyn Pirie, Gillian Councill

**Affiliations:** ^1^NHS Grampian, Aberdeen, United Kingdom; ^2^Alzheimer Scotland, Aberdeen, United Kingdom

## Abstract

**Aims:** Brain Health Scotland (BHS) is a pioneering nationwide initiative aimed at preventing, assessing, and treating dementia. A collaboration between the Scottish Government (SG), Alzheimer Scotland (AS) and NHS Public Health, the service supports people of any age to understand dementia risk factors, provides early diagnosis, and offers brain health personalised action plans. Aberdeen hosts a 2 year pilot of a Brain Health Service in Scotland, with co-location of AS and NHS Grampian Public Health staff (Psychiatrist/Mental Health Nurse) in a city centre AS building. A unique feature is a self-referral route for people concerned about memory function. Stage 1 of the service involves a risk discussion with an advisor and the provision of a Personalised Action Plan. If the service-user reports memory impairment they enter Stage 2 (Nursing assessment, including ACE) and if more detailed assessment is required they enter Stage 3 (Psychiatrist). GPs can refer patients with mild memory impairment to Stage 2–3. A SG commissioned independent evaluation has issued an interim report.

**Methods:** We analysed qualitative and quantitative data from the project’s first year including service-user comments in the evaluation interim report and data from an audit of NHS patients.

**Results:** 142 people accessed the service (99 by self-referral) and received a brain health assessment, including evaluation of risk factors for dementia and provision of a personalised action plan. 102 people reported memory impairment and were seen in the NHS clinic. The average age was 64 and the average Addenbrooke’s (ACE) score was 87. 29 patients had Mild Cognitive Impairment, 21 had psychological difficulties, 6 had dementia, substance misuse and severe mental illness respectively and the others had a wide range of problems including Motor Neurone Disease (n=1), Lyme disease (n=1), Functional Cognitive Disorder (n=1) and ADHD (n=1). Patients were referred for investigations such as brain scans (including CT, MRI, SPECT) and Neuropsychological assessment as appropriate.

**Conclusion:** The Aberdeen Brain Health Service demonstrates excellent collaboration between the NHS and 3rd Sector and incorporates Public Health and Mental Health principles. Service-user satisfaction is high for all stages of the service. An independent evaluation will inform discussions with the SG about potential service models of Brain Health Services across Scotland.

